# Salivary Detection of Dengue Virus NS1 Protein with a Label-Free Immunosensor for Early Dengue Diagnosis

**DOI:** 10.3390/s18082641

**Published:** 2018-08-12

**Authors:** Daniel Wasik, Ashok Mulchandani, Marylynn V. Yates

**Affiliations:** 1 Department of Environmental Sciences, University of California, Riverside, CA 92521, USA; dwasi001@ucr.edu (D.W.); marylynn.yates@ucr.edu (M.V.Y.); 2 Department of Chemical and Environmental Engineering, University of California, Riverside, CA 92521, USA

**Keywords:** dengue virus, non-structural 1 (NS1) protein, label-free biosensor, immunosensor, carbon nanotubes, saliva

## Abstract

Dengue virus (DENV) is a highly pathogenic, arthropod-borne virus transmitted between people by Aedes mosquitoes. Despite efforts to prevent global spread, the potential for DENV epidemics is increasing world-wide. Annually, 3.6 billion people are at risk of infection. With no licensed vaccine, early diagnosis of dengue infection is critical for clinical management and patient survival. Detection of DENV non-structural protein 1 (NS1) is a clinically accepted biomarker for the early detection of DENV infection. Unfortunately, virtually all of the laboratory and commercial DENV NS1 diagnostic methods require a blood draw for sample analysis, limiting point-of-care diagnostics and decreases patient willingness. Alternatively, NS1 in human saliva has been identified for the potential early diagnosis of DENV infection. The collection of saliva is simple, non-invasive, painless, and inexpensive, even by minimally trained personnel. In this study, we present a label-free chemiresistive immunosensor for the detection of the DENV NS1 protein utilizing a network of single-walled carbon nanotubes functionalized with anti-dengue NS1 monoclonal antibodies. NS1 was successfully detected in adulterated artificial human saliva over the range of clinically relevant concentrations with high sensitivity and selectivity. It has potential application in clinical diagnosis and the ease of collection allows for self-testing, even within the home.

## 1. Introduction

Dengue virus (DENV) is an arthropod-borne flavivirus primarily transmitted by the mosquito vectors *Aedes aegypti* and *Aedes albopictus* [1]. Prior to World War II, the mosquito vectors, and therefore the virus, were mostly limited to tropic and sub-tropic regions. However, since the 1950s, increased air travel, global commerce, unplanned urbanization, and global warming have permitted the mosquito vectors to proliferate in previously unaffected regions [2]. Autochthonous infections have now been reported as far north as France, Japan, and the USA; indicating that DENV is a growing global threat [3,4,5].

Dengue fever, the most frequent result of a DENV infection, has the highest incident rate among humans of any of the arboviral diseases. It has recently been estimated that 390 million new infections occur annually [6] and 3.6 billion people are at risk of infection [7], with the highest rates of infection occurring among children who are 15 years of age or younger [8]. The clinical manifestations of dengue fever vary from asymptomatic to severe arthralgia and myalgia, with typical infections manifesting as a nonspecific febrile disease. In some cases, the infection causes severe dengue, which can result in failure of the circulatory system, the liver, and death if not adequately managed [9].

There are five antigenically distinct dengue viruses, DENV1-DENV5, with each capable of causing dengue fever and severe dengue [10]. There is no specific treatment for a DENV infection; however, early intervention with fluid replacement therapy can reduce mortality from 20% to below 1% [1]. Therefore, diagnosis of a dengue infection is critical for clinical management, especially when late or inadequate treatment can be lethal. Unfortunately, dengue and severe dengue have no pathognomonic clinical features and can manifest differently in adults and children, making a clinical diagnosis and differentiation of dengue or severe dengue by clinical features alone extremely difficult [9,11].

A number of diagnostic tests have been produced to aid in clinical diagnosis by detecting the virions, nucleic acids, serologic, or antigenic components of a DENV infection. Viral isolation by culture and nucleic acid detection with polymerase chain reaction-based techniques require a dedicated laboratory, expensive equipment, and highly trained personnel, which are impractical for routine clinical diagnostics [12]. Current commercially available rapid diagnostic tests (RDTs) are relatively inexpensive, easily accessible by untrained personnel, and they employ various technologies for serological or antigenic detection. Serologic RDTs detect the IgG and IgM antibodies produced during a DENV infection. Unfortunately, the developed IgG and IgM are not always highly specific to DENV, and serological assays are thus known to be cross-reactive against other flaviviruses [13,14]. Additionally, it can take up to 7 days post-infection for antibody concentrations to reach detectable limits [1,8], and the antibodies from any flavivirus infection stay in the blood for months, triggering future false positives [9,12]. Antigen-detecting RDTs typically use a lateral flow or ELISA-based methods for detection of the highly conserved DENV non-structural protein 1 (NS1). NS1 is a 46-kDa protein secreted by infected cells, has a clinical range from 0.04 to 2 µg/mL in human serum, and can be detected within the first 18 days of a primary infection [15]. NS1 is an ideal target for the early detection of a DENV infection, and high NS1 concentrations and/or a rapid decline of soluble NS1 may be an indicator of a severe DENV infection [16,17]. However, current commercially available lateral flow and ELISA-based RDTs do not quantify NS1 concentrations, and sensitivities under 50% have been reported during the first two days of illness, when serum NS1 concentrations are low [18,19]. Importantly, each of these diagnostic assays require blood to be drawn, ranging from capillary to venous collection, for viral diagnosis and laboratory confirmation using whole blood, serum, and/or plasma samples. Venous blood collection requires expertise and equipment typically found in a clinical setting but can be a limiting factor for in-home testing or for use by volunteers or workers with limited training. Additionally, the blood draw also exposes personnel to bloodborne pathogens and needle-stick injuries; and are sources of pain for both adults and children, leading to an unwillingness to agree to such procedures [20,21,22,23].

Alternatively, saliva collection is relatively non-invasive, painless, inexpensive, and simple to collect, even by minimally trained personnel. Whole saliva is produced by a continuous flow of water, mucins, and enzymes generated by salivary glands as well as serumnal components transported from the blood into saliva [24,25]. The relationship between NS1 concentrations in saliva and blood is not well understood; preliminary studies have identified NS1 detection in saliva as a diagnostic possibility and have shown that saliva NS1 concentrations correlate with DENV-RNA serum levels [26,27,28,29]. Andries et al. [29] reported that in a cohort of 267 patients aged 3–16, NS1 in saliva was detectable up to two weeks after the onset of fever. NS1 concentrations in saliva samples obtained by direct spitting were an average of 3.8 ng/mL and ranged between 0.5 ng/mL and 41.5 ng/mL; too low for reliable diagnosis with commercially available RDTs [18,19].

Electronic biosensors fabricated with single-walled carbon nanotube (SWNT) transducer elements make ideal detection tools when ultra-low protein concentrations are expected [30,31,32]. SWNTs have a high surface-to-volume ratio for functionalization with antibody bioreceptors (immunosensors) that enable selective and specific detection of target analytes. The 1D structure of the SWNT allows small perturbations of antibody-analyte binding events to be converted into a measurable electric signal, even at low analyte concentrations and without labelling. Additionally, these immunosensors are portable, rapid, and inexpensive diagnostic tools suitable for in-home and point-of-care use.

In this study, we report a chemiresistive immunosensor for the label-free detection and quantification of dengue NS1 in artificial saliva. Anti-dengue NS1 monoclonal antibodies were functionalized onto a dense network of self-assembled SWNT on lithographically patterned interdigitated gold microelectrodes. The immunosensor performance was assessed in phosphate buffer (PB) and artificial human saliva where it demonstrated quantification of DENV NS1 protein concentrations with high sensitivity and specificity.

## 2. Materials and Methods

### 2.1. Fabrication of SWNT Immunosensors

#### 2.1.1. Microelectrode Fabrication

Photolithography and e-beam evaporation was used to form the microelectrodes by depositing a 20-nm Ti adhesion layer and a 180-nm Au gold contact layer onto the 300-nm SiO_2_ layer of a silicon wafer [33]. Each microelectrode has single sensor consisting of 10 pairs of 5 μm wide interdigitated fingers separated by a 3-μm gap. A chip has five independent microelectrodes with the sensors located in close proximity to one another, forming a sensing region. Each chip was bathed in 30% ammonium hydroxide (Fisher Scientific, Inc., Hampton, NH, USA, A669-212) for 30 min and rinsed with deionized water (DI). Next, the sensing region was covered by 3-aminopropyltriethoxysilane (APTES) (Acros Organics, Fair Lawn, NJ, USA, 99%) for 30 min followed by washing in DI. A 10 μL drop of 0.01 mg/mL of a 95% semiconductive SWNT suspension (Nanointegris Inc., Boisbriand, QC, Canada) was dropcasted onto the sensing region for 1 h followed by annealing for 1 h at 250 °C in an open-ended quartz tube resulting in a self-assembled network of SWNT. Annealed chips were stored under vacuum until use.

#### 2.1.2. Preparation of Anti-Dengue Virus NS1 Glycoprotein Antibody and NS1 Glycoprotein

Micro Bio-Spin P-6 Gel Columns in 10 mM Tris–HCl buffer, pH 7.4 (732-6222, Bio-Rad, Hercules, CA, USA) were used to buffer exchange mouse monoclonal anti-dengue virus NS1 glycoprotein antibodies (ab138696, Abcam, Cambridge, UK) into 10 mM phosphate buffer, pH 7.2 (PB) according to the manufacturer’s instructions. After buffer exchange, antibody concentration was 240 μg/mL. Similarly, full-length dengue virus NS1 glycoprotein (ab64456, Abcam) was buffer exchanged into PB for a final concentration of 0.1 mg/mL.

#### 2.1.3. Preparation of Adulterated PB and Artificial Human Saliva Samples

Artificial human saliva was prepared as previously described in Tilli et al. [32] with 0.6 g/L Na_2_HPO_4_, 0.6 g/L anhydrous CaCl_2_, 0.4 g/L KCl, 0.4 g/L NaCl, 4 g/L mucin, and 4 g/L urea dissolved in DI. The mixture was adjusted to pH 7.2 and then sterilized by autoclaving and kept at 4 °C until use. Buffer exchanged DENV NS1 protein was pipetted into an aliquot of artificial saliva and then serially diluted into other saliva aliquots. The NS1 adulterated artificial saliva samples were immediately clarified by centrifugation at 14,000 × *g* for 5 min at 4 °C. The supernatant was extracted and kept on ice for immediate analysis; the pellet was discarded. PB samples adulterated with NS1 were similarly prepared.

#### 2.1.4. Functionalization of SWNT Networks with Anti-Dengue Virus NS1 Glycoprotein Antibody

**Figure 1:**
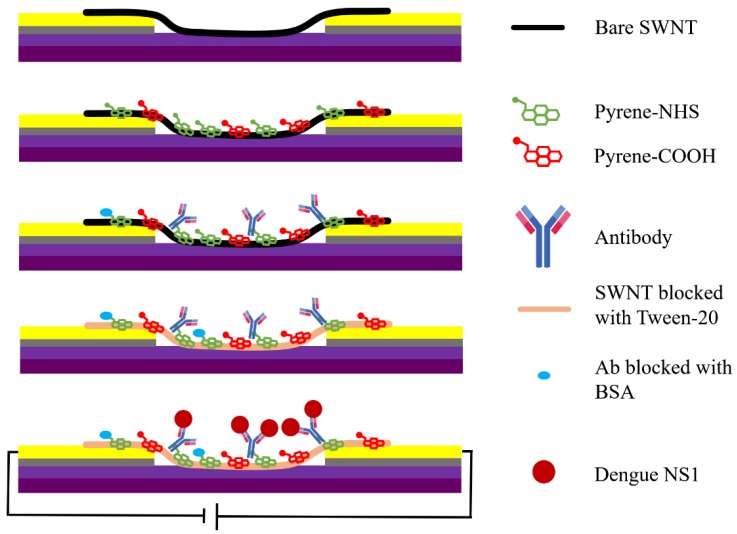
Schematic of functionalization of single-walled nanotubes (SWNTs) networks with anti-non-structural protein 1 (NS1) antibody.

Figure 1 shows a schematic of the functionalization of the SWNT networks with anti-NS1 antibody. In brief, the chips were placed in a chamber with humidity controlled by petri dish containing DI water. The SWNT networks are first incubated for 1 h with a mixture of 1-Pyrenebutyric acid (Sigma Aldrich, St. Louis, MO, USA) and 1-Pyrenebutyric acid N-hydroxysuccinimide ester (PBASE) (Sigma Aldrich, St. Louis, MO, USA) at a 3:1 molar ratio into 1 mL of dimethylformamide (DMF). This resulted in the pyrene moieties non-covalently attaching to the SWNT by π-π stacking while exposing the N-hydroxysuccinimide ester for covalent attachment with anti-NS1 antibody with an amide bond [34,35]. Chips were washed with DMF, followed by drying with N_2_ gas. Each of the following incubation steps were in the dark at 4 °C with similarly controlled humidity. The entire surface of the chip was covered by 2 mM 6-mercaptohexanol in DMF for 30 min followed by washing with DMF. Next, a 10 μL drop of buffer-exchanged antibody was pipetted onto the sensing region and incubated for 2 h, creating five independent immunosensors on a single chip. Finally, in order to block non-specific interactions with the SWNT or antibodies, the immunosensor was incubated with 0.1% Tween 20 in DI (BIO-RAD) for 1 h, gently washed with PB, and then 1% bovine serum albumin (BSA) for 1 h followed by gentle washing with PB.

### 2.2. Detection of the NS1 Protein

Measurements were performed in the dark with high humidity. The resistance of the five immunosensors per chip was individually measured at a fixed source-drain potential (VDS) of 0.1 V by a Keithley 2636 SourceMeter (Tektronix, Beaverton, OR, USA). As a control, two incubations with unadulterated PB or artificial saliva performed prior to sample analysis, immunosensors with ≥ ±5% change in normalized resistance were excluded. During incubation, the sensing region was covered by 10 μL of sample solution for 10 min. The sample solution consisted of unadulterated PB, unadulterated artificial saliva, PB adulterated with NS1 or BSA, or artificial saliva adulterated with NS1 or BSA. Unadulterated samples were used for blanks and control measurements, and adulterated samples were used for detection and control measurements. The incubation of a sample solution was immediately followed by gentle washing and a 20 μL drop of PB to cover the sensing region. To detect the newly bound analyte from sample incubation, the resistance was permitted to stabilize under the 20 μL drop of PB and a resistance measurement was taken. Sample concentration started at the lowest concentration and was increased step-wise with 10-fold increments. All measurements were made at room temperature (~22 °C). Following the highest NS1 concentration, another incubation and measurement using the blank solution was performed. The normalized resistance change of each immunosensor (Equation (1)), was calculated using the equation

[(R_1_ − R_0_) × 100/R_0_] (1)

where, R_1_ is the stabilized resistance of the immunosensor in PB and R_0_ is the stabilized resistance measurement taken under the 20 μL drop of PB. The result is a percent resistance change that is normalized to the blank reading.

## 3. Results and Discussion

**Figure 2:**
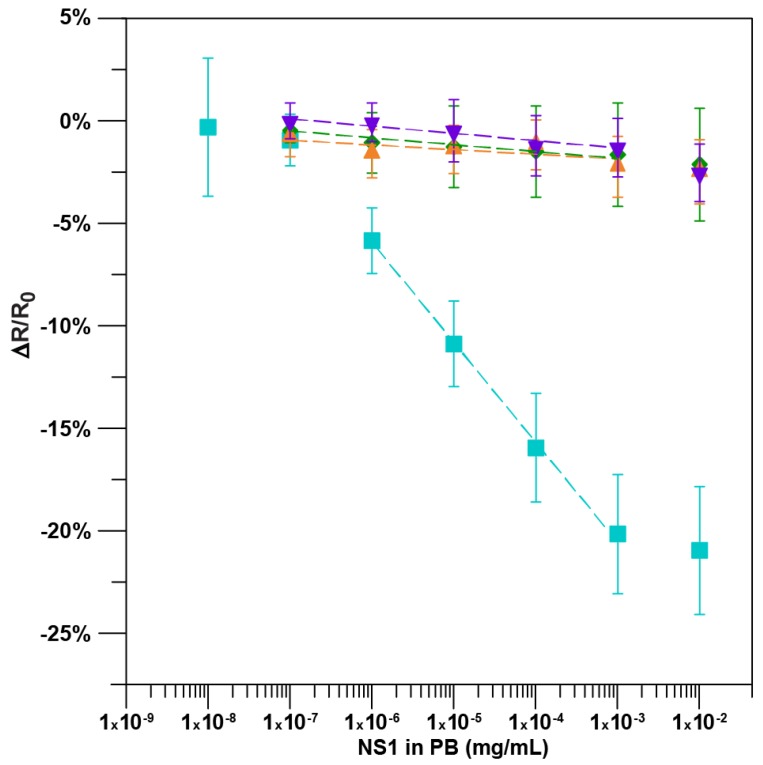
Immunosensor calibration plot: normalized response of the immunosensors functionalized with bovine serum albumin (BSA), Tween-20, anti-NS1 antibody after incubation with NS1 protein in PB [*n =* 7; y = −4.88x − 35.23, *R*^2^ = 0.99] (■), after incubation with PB only [*n =* 6, y = −0.36x − 2.4, *R*^2^ = 0.91] (▼), and after incubation with BSA protein in PB [*n =* 9; y = −0.24x − 2.6 *R*^2^ = 0.53] (▲). Normalized response of the immunosensors functionalized with BSA and Tween-20 but without antibody to the incubation of NS1 protein in PB [*n =* 8; y = −0.34x − 2.9, *R*^2^ = 0.93] (♦). The data points are the mean of measurements from independent immunosensors, and error bars represent ±1 standard deviation.

The calibration curve in Figure 2 was generated by averaging the normalized percent resistance change for each sample incubation. A positive control in which biosensors without an anti-NS1 antibody were challenged with increasing concentrations of NS1 in PB and a negative control in which devices with antibody were challenged with PB alone resulted in minimal average change of normalized resistance per incubation: −0.34 ± 0.05% and −0.36 ± 0.06%, respectively. When an immunosensor with anti-NS1 antibody was incubated with PB adulterated with BSA, the average normalized change in resistance was −0.24 ± 0.13% per incubation. The three controls were not significantly different from each other (*n =* 23, *p* > 0.05). The average response from the incubation of increasing concentrations of NS1 in PB from 0.1 ng/mL NS1 up to 10,000 ng/mL NS1 is presented. The 1 ng/mL of NS1 in PB was the lowest concentration of PB adulterated with NS1 that was significantly greater than the controls (*n =* 7, *p* < 0.01) and resulted in a −5.89 ± 1.6% decrease in resistance. The immunosensor calibration plot for NS1 in PB was linear between 1.0 ng/mL and 1000.0 ng/mL (y = −4.88x − 35.23, *R*^2^ = 0.99; *n =* 7) with a sensitivity (slope) of −4.88 ± 0.13% per log ng/mL. The increase in resistance to NS1 additions is likely due to the electron scattering/donation potential onto semiconducting *p*-type SWNT [33,36,37].

The mucus of uncentrifuged artificial human saliva left behind a visible residue on the sensor that was not easily removed even by perfuse washing with buffer. The residue caused the resistance of the immunosensor to remain relatively high, preventing NS1 detection. To eliminate the interference, the artificial saliva was centrifuged using established protocols [24,28,38]. Centrifuged saliva samples did not leave behind any visible residue and the immunosensor returned to a relatively lower resistance in which NS1 detection and quantification was possible.

**Figure 3:**
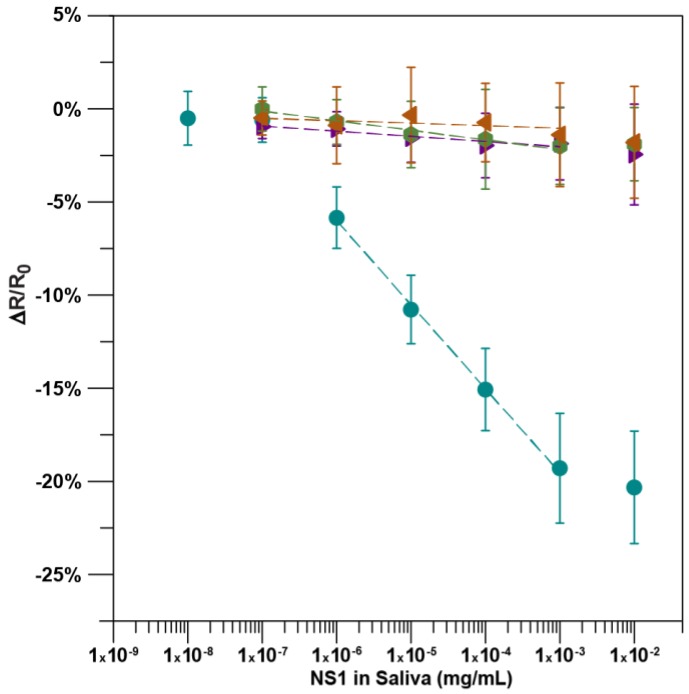
Immunosensor calibration plot: normalized response of the immunosensors functionalized with BSA, Tween-20, and anti-NS1 antibody after incubation with NS1 protein in artificial human saliva [*n =* 17; y = −4.79x − 34.3, *R*^2^ = 0.99] (●), after incubation with saliva only [*n =* 6, y = −0.28x − 2.9, *R*^2^ = 0.92] (►), and after incubation with BSA protein in artificial saliva [*n =* 7; y = −0.14x − 1.4, *R*^2^ = 0.48] (◄). Normalized response of the immunosensors functionalized with BSA and Tween-20 but without antibody to the incubation of NS1 protein in artificial saliva [*n =* 5; y = −0.52x − 3.7, *R*^2^ = 0.96] (●). The data points are the mean of measurements from independent immunosensors, and error bars represent ±1 standard deviation.

As shown in Figure 3, the average response of the immunosensors exhibits a sigmoidal curve to increasing concentrations of NS1 in artificial human saliva. Similar to NS1 in PB, the lowest concentration of NS1 in artificial saliva to be significantly different from that of the controls was 1.0 ng/ml NS1, with −5.85 ± 1.67% change in resistance (*n =* 17, *p* < 0.05). At the highest NS1 concentration possible, 10,000 ng/mL in PB (not artificial saliva), the normalized resistance only changed by −1.15 ± 1.34% relative to the previous incubation giving an approximate upper limit of quantification of 1000 ng/mL NS1 in artificial saliva. Through the quantification range, the resistance decreased linearly (y = −4.52x − 33.1, *R*^2^ = 0.998; *n =* 17) as a function of the logarithm of NS1 concentration. The immunosensor had a sensitivity of −4.52 ± 0.13% per log ng/mL. A terminal unadulterated artificial saliva incubation resulted in a minimal 0.08 ± 1.3% average change in resistance across the same immunosensors, demonstrating that resistance changes were not a result of repeated challenges or the washing of the sensor. The immunosensors exhibited a minimal response to artificial saliva adulterated with BSA and unadulterated saliva, with a −0.14 ± 0.09% and 0.27 ± 0.05% average change in resistance per incubation, respectively. Additionally, when sensors without antibody were challenged with artificial saliva adulterated with increasing concentrations of NS1, the normalized sensor response was −0.52 ± 0.06% per incubation, which was not significantly different to the other controls at any concentration. (*n =* 5, *p* > 0.05) All three controls were not significantly different from each other, indicating that the functionalized immunosensor is highly selective and the resistance change is due to the capture of DENV NS1 by the anti-NS1 antibody (*n =* 18, *p* > 0.05).

The label-free immunosensor presented in this work detected DENV NS1 in concentrations as low as 1 ng/mL with only a 10 min incubation of adulterated artificial saliva. Radzol et al. [39] reported detection of NS1 in saliva using surface enhanced Raman spectroscopy (SERS) at concentrations as low as 10 ng/mL [28,40]. However, SERS requires expensive equipment and expertise not readily available in the point-of-use environment. On the other hand, Andries et al. [29] used saliva samples obtained by direct spitting and was able to detect NS1 in whole saliva down to 0.5 ng/mL using a relatively simple one-step ELISA. However, this required an incubation of 2 h, the collection of large sample volumes, and labeled antibodies. In a separate study, Anders et al. [41] tested oral swabs with the Bio-Rad NS1 Platelia ELISA, with a limit of detection of 11.94 ng/mL [42], to detect NS1 in 64.7% patients (*n =* 85) that tested plasma positive. Finally, in another study, Andries et al. [43] found that a “prototype immunochromatographic kit” developed by Standard Diagnostics for detection of NS1 in whole human saliva lacked sensitivity and specificity (33.9%, *n =* 59). Similar to the experiment presented here, the viscous mucin present in human saliva was reported to have interfered with NS1 detection and caused the “prototype immunochromatographic kit” to have a lower sensitivity [43]. Interference by mucin and other salivary components inherent to saliva are known problems for salivary diagnostic testing [44], prompting the exploration of methods to eliminate these compounds without the need for a centrifugation pre-treatment [45]. Furthermore, NS1 concentration in a saliva sample appears to be greatly affected by the collection technique, with direct spitting obtaining a higher concentration [29]. The complexity and production of saliva can be impacted by a number of factors during sample collection; thereby resulting in changes in the concentration of target biomarkers [46]. For example, the use of a cotton swab for saliva collection may have significantly interfered with collection of the DENV NS1 protein and resulted in a lower NS1 concentration in the analyzed saliva [47]. The challenges associated with the collection of saliva without effecting the concentration of the desired biomarker is currently being explored [48,49,50]. Clearly, more studies are needed to establish which saliva collection technique is most effective for NS1 detection.

High concentrations of NS1 in serum and possibly saliva may be an indicator of severe dengue [51,52]. While the association between NS1 concentration and severe dengue appears to correlate with circulating DENV serotypes, clinicians commonly rely on NS1 antigen testing of drawn blood samples for early detection of a DENV infection. Unfortunately, this method necessitates well-trained personnel, is risky and unattractive to patients, especially children, and NS1 concentrations may be too low during the earliest and later stages of infection for RDTs, often resulting in false negatives [53].

## 4. Conclusions

Unlike RDTs [52], the presented immunosensor is compatible with saliva collection, a non-invasive sampling technique that is much more likely to be accepted by patients and can be collected by untrained personnel. With a quantification range of ~1 ng/mL to 1000 ng/mL of NS1 in artificial human saliva, the proposed biosensor is capable of quantifying DENV NS1 in a clinically relevant salivary concentration range with a 10 min incubation of 10 μL of saliva. As such, the immunosensor will improve clinical utility and diagnostics in the point-of-care setting, especially when a blood draw is unavailable, and may distinguish dengue from severe dengue. The ease of collection and analysis permits self-testing by a potentially infected individual within their home.
